# Supplementary material for: A QUBO formulation for top-τ eigencentrality nodes
Source: PLoS One. 2022 Jul 14;17(7):e0271292. doi: 10.1371/journal.pone.0271292 (PMC9282604; doi:10.1371/journal.pone.0271292)
Supplement: S1 Appendix — (PDF) [file pone.0271292.s001.pdf]

Supporting information

S1 Appendix. Computing degree centrality and eigencentrality from exponential function.

Consider the exponential function defined by

$$f(x) = e^x = \sum_{k=0}^{\infty} \frac{x^k}{k!} \tag{13}$$

Since f is continuous and analytic with radius of convergence infinity, we observe that for a primitive matrix A which is mostly the case for the adjacency matrix of simply connected undirected graphs

$$f(\gamma A) = e^{\gamma A} = \sum_{k=0}^{\infty} \frac{(\gamma A)^k}{k!} \tag{14}$$

Then $f(\gamma A)$ is a matrix and the components of the vector $f(\gamma A)\mathbf{1}$ where $\mathbf{1}$ is a vector of ones, counts the walks of infinite length centered at each node. Let $\{\boldsymbol{e_i} \in \mathbb{R}^n\}$ be the

set of canonical basis vectors for $\mathbb{R}^n$ whose only nonzero element is the ith component.

$$
\begin{aligned}
v_i(\gamma) &= \sum_{j=0}^{n-1} \boldsymbol{e_i}^T f(\gamma A)\boldsymbol{e_j} \\
&= \sum_{k=0}^{\infty}\sum_{j=0}^{n-1} \boldsymbol{e_i}^T \frac{(\gamma A)^k}{k!}\boldsymbol{e_j} \\
&= \sum_{k=0}^{\infty}\sum_{j=0}^{n-1} a_k \gamma^k \boldsymbol{e_i}^T A^k \boldsymbol{e_j} \\
&= a_0 \sum_{j=0}^{n-1} \boldsymbol{e_i}^T \boldsymbol{e_i} + a_1\gamma \sum_{j=0}^{n-1} \boldsymbol{e_i}^T A\boldsymbol{e_j} + \ldots \\
&= a_0 + a_1\gamma d_i + \sum_{k=0}^{\infty}\sum_{j=0}^{n-1} a_k \gamma^k \boldsymbol{e_i}^T A^k \boldsymbol{e_j} \\
c_i(\gamma) = \frac{v_i(\gamma) - a_0}{a_1\gamma} &= d_i + \sum_{k=2}^{\infty}\sum_{j=0}^{n-1} \frac{a_k}{a_1}\gamma^{k-1} \boldsymbol{e_i}^T A^k \boldsymbol{e_j} \qquad (15)
\end{aligned}
$$

where d_i is the degree of the ith node. In the limit $\gamma \longrightarrow 0^+$, Eq. (15) converges to the degree centrality and by expanding in the eigenbasis, it converges to EC for $\gamma \longrightarrow \infty$ [10].
