# Supplementary material for: A QUBO formulation for top-τ eigencentrality nodes
Source: PLoS One. 2022 Jul 14;17(7):e0271292. doi: 10.1371/journal.pone.0271292 (PMC9282604; doi:10.1371/journal.pone.0271292)
Supplement: Attachment — Submitted filename: Response to Reviewers.pdf [file pone.0271292.s002.pdf]

Computer, Computational, and Statistical Sciences Division,
Los Alamos National Laboratory,
Los Alamos, New Mexico, United States, 87545

(505) 667-0790
smm@lanl.gov

Dear Editor,

Please find attached a revised version of the manuscript entitled "A QUBO formulation for top-τ eigencentrality nodes" by Prosper D. Akrobotu, Tamsin E. James, Christian F. A. Negre, and Susan M. Mniszewski, for publication as a research paper in PLOS ONE.

All revisions have been addressed and a point-by-point response to the comments of the reviewers is attached. Most importantly, new paragraphs have been added in order to clarify the points made by the reviewers.

At this point, we hope the revised manuscript is acceptable for publication, but also remain open to providing additional specific revisions. We would like to thank the reviewers for their helpful comments and your editorial office for your work and consideration.

With best regards,

Prosper D. Akrobotu
Susan M. Mniszewski

Response to the reviewers

We are thankful to the anonymous reviewers for their helpful comments and have prepared a detailed response below. Each referee's comments are numbered and quoted in ***bold italic*** typeface, our replies can be found below each comment in roman typeface. New sections added to the manuscript are written in *red italic* typeface.

Reviewer 1

1. ***Claims are not clear enough.***
- What exactly is the problem being solved? The text, in some places, seems to focus on the problem of computing eigencentrality scores or at least is not clear enough on what the problem being solved. For example, "we lay the foundation for the calculation of eigenvector centrality using quantum ...." or "Eigenvector centrality as a QUBO problem" is different than a formulation that identify the top N most central users. While it can be run repeatedly to generate ranking, this is a much more complex procedure, and still does not actually compute the scores but the ranking. This seems to also impact the presentation of the approach: instead of a clear problem definition, the paper presents the original problem of eigencentrality calculation and transforms it into a different problem that can be formulated using QUBO. I think the paper would benefit from a clear problem definition (i.e., identifying a given number of most central users) and then a QUBO formulation that solves this problem. - As there is no clear problem definition, the paper also does not provide a clear statement on the connection between the proposed solution and the problem being solved. For example, it is not clear if the optimal solution for the QUBO formulation (assuming it is found) is guaranteed to be the optimal solution for the problem (i.e., the given number of most central nodes) or is a heuristic approach that seems to work well in practice (see related points on Proposition 1 and Claim 1 below).

    - We agree with the reviewer. Our objective is not to compute the eigenvector centrality scores but rather the most central nodes withi a given graph using the eigenvector centrality as a metric. In consequence we decided to change the title to relate directly with the problem being solved as well as to define a

clear problem statement.

New title: *A QUBO formulation for top-τ eigencentrality nodes*

As networks become larger and more complicated, the efficient calculation of the centrality or "hierarchy" of nodes in a network becomes more relevant in network analysis for decision making. Decision makers are usually interested in determining just a fraction of the most central nodes in the network due to limited resources or budgetary constraints. For example, in the COVID-19 pandemic, the Centers for Disease Control and Prevention (CDC), limited by inadequate vaccines, might be interested in identifying only a fraction of the population that greatly influence the spread of disease or safe spots to quarantine people. Thus, identifying the top-τ most influential nodes is an essential inevitable problem to address in network analysis. The world is likely to embrace quantum computing in the near future and hence understanding and developing quantum computing formulations of this problem considering EC of nodes would be a major advance in the field of quantum computing. Our research, thus, seeks to address this problem by reformulating the problem of identifying the top-τ most influential nodes (highest EC nodes) as a quadratic unconstrained binary optimization (QUBO) problem to be solved on quantum computers.....

The main goal of this paper as stated earlier,is to develop a QUBO formulation for the problem of identifying the top-τ highest EC nodes, and implement it on quantum annealing and gate-based quantum computers. Since EC is also a good measure for generating node ranks we also explore the possibility of our formulation generating node ranks.

- Since we are not computing the centrality scores, we have also changed the title of the subsection "Eigenvector centrality as a QUBO problem" to
  Top-τ most influential nodes as a QUBO problem
- Since the QUBO was formulated by modifying the actual problem Hamiltonian $(A - \lambda I)^2$ without any rigorous mathematical proof, the method presented is simply a heuristic approach that seems to work well in practice based on the experiments we conducted. To justify if the solution to the QUBO problem is guaranteed to be the optimal solution demands a rigorous mathematical proof which is open for our future study.

2. ***No sufficient theoretical support for proposed approach and claims: - No proof for proposition 1, and there isn't a more general proof for the case of $\tau > 1$ (which seems to be formulated in Claim 1). The paper mentions the authors were able to "verify for proposition 1 (with $\tau >= 1$) for ..." however these are empirical results and not a proof. - There are no theoretical results on sufficient/optimal selection of P values (the Lagrange multiplier). This is also related to the previous point as I was surprised that proposition 1 does not include any conditions on the P values (e.g., is it still correct when P_0 or P_1 are equal to zero?). Also, there is no principled explanation/justification for the values used in the experiments.***

   - The reviewer is right, our presentation and justification is more experimental and has no formal theoretical proof and hence we eliminate the proposition 1 and redefine it as an optimization problem we want to solve using approximate methods.

     We proceed by solving for and examining the ground state of the QUBO problem

     $$\begin{aligned} &\min_{\boldsymbol{x}\in\{0,1\}^n} \boldsymbol{x}^T Q \boldsymbol{x} \\ &Q = -P_0 A^2 \hat{\boldsymbol{d}}\hat{\boldsymbol{d}}^T A - P_0 A \hat{\boldsymbol{d}}\hat{\boldsymbol{d}}^T A^2 + P_1 C \\ &C = (1-2\tau)I + U \\ &P_1 > P_0 > 0 \end{aligned} \tag{P}$$

     with $\tau = 1$, where the matrix U .....

   - *....we performed several experiments (with $\tau \geq 1$) on the D-Wave 2000Q/IBM-Q machines for different graphs (see Fig. 2 and Fig. 3). We observed that the solution to the QUBO problem P strongly correlates with the top-τ most important EC nodes when compared to the solution from the power method of NetworkX's EC algorithm.*

   - Although there is no theoretical proof, our approach follows the principle of quantum search by continuous time quantum walk algorithms.

     The form of our problem Hamiltonian Q was chosen to mimic the search Hamiltonian, $H = -\gamma L - \boldsymbol{w}\boldsymbol{w}^T = \gamma(A - D) - \boldsymbol{w}\boldsymbol{w}^T$, for quantum search by a continuous time quantum walk algorithm for a marked node w in a graph described by Childs

and Goldstone in [1]. Here, the matrix L represents the graph's Laplacian which is the difference between the graph's adjacency matrix A and the diagonal matrix D of degrees of the nodes. The principle behind this quantum search is that the evolution of the Hamiltonian H in a time inversely proportional to the energy gap $(E_1 - E_0)$ between the ground and first excited state energy E_0 and E_1 respectively generates a rotation between a start state $\boldsymbol{s} = \frac{1}{\sqrt{N}} \sum_{i=1}^{n} \boldsymbol{e_i}$, a superposition of all the states, and a state with a significant overlap with the marked state $\boldsymbol{w}$ provided the first excited state has a significant overlap with the states $\boldsymbol{s}$ and $\boldsymbol{w}$ over $\gamma \in (0, \infty)$ [1]. Observing that the first excited state of the minimization problem of the objective function Eq. (10) always had a significant overlap with our desired solution of the top-τ most influential nodes, we examined each term of Eq. (10) and constructed a symmetric matrix from the outer product of the vectors $A^2\boldsymbol{d}$ and $A\boldsymbol{d}$ to get $A^2\boldsymbol{d}(A\boldsymbol{d})^T + A\boldsymbol{d}(A^2\boldsymbol{d})^T = A^2\boldsymbol{d}\boldsymbol{d}^T A + A\boldsymbol{d}\boldsymbol{d}^T A^2$. The vector $\boldsymbol{d}$ is introduced because of the factor d_{max} in Eq. (10) We proceed by solving for and examining the ground state of the QUBO problem

$$\begin{aligned} &\min_{\boldsymbol{x} \in \{0,1\}^n} \boldsymbol{x}^T Q \boldsymbol{x} \\ &Q = -P_0 A^2 \hat{\boldsymbol{d}}\hat{\boldsymbol{d}}^T A - P_0 A \hat{\boldsymbol{d}}\hat{\boldsymbol{d}}^T A^2 + P_1 C \\ &C = (1 - 2\tau) I + U \\ &P_1 > P_0 > 0 \end{aligned} \tag{P}$$

with $\tau = 1$, where the matrix U is defined in Eq. (9).

- The value $P_0 = \frac{1}{\sqrt{n}}$ is chosen because the value $\gamma = \frac{1}{\sqrt{n}}$ was found to be the optimal valued in the quantum search by continuous time quantum walk [1].

3. ***The empirical support could be strengthened and some important details on the experiments should be clarified: - As mentioned earlier, many results are only verified experimentally and not theoretically. Since all the graphs used are very small and in a limited range of values, it is not clear how the results will change as graphs become larger (e.g., should the P_0 and P_1 values used remain the same?). While the limited size is justified by the hardware limitations, larger graphs could potentially be tested on simulators. - The paper mentions that in some graphs there was a need for multiple runs to capture global minimum. How many runs were used and how was***

it determined if the global minimum was captured or additional runs are needed? - Why is there no result (or "-") in the "top 5 most central nodes" for QA in the BA(50,5) graph? - The paper should list and cite the concrete algorithms being used and not just the libraries, e.g., which algorithm (that is implemented in NetworkX) is used for computing EC? which classical optimization algorithm (that is implemented in SciPy) was used? - It was not clear to me what claim is supported by the analysis of the performance of SciPy in Table 2 (related to point (1) above)

- Based on experimental results, the P_0 and P_1 values can be varied for larger graphs, however we were more interested in using a more generic expression for P_0 and P_1 and hence settled on the chosen expressions which worked for all graphs considered.
- Using the same values for P_0 and P_1 we experimented on the QUBO for our largest graph, BA(50,5) graph using simulated annealing with different values for $\tau \leq 10$ and observed results matching those from the tabu QBSolv and the power method of NetworkX.
- *The minimum solution obtained is affirmed global minimum when it's energy equals that of the exact classical Numpy Minimum Eigensolver algorithm (for implementations on IBM-Q) or tabu QBSolv (for implementations on D-Wave 2000Q).*
- *...there is no fixed number of runs determined for all graphs to guarantee the global minimum solution. For example, in Fig. 1 we see that QA returns the global minimum for QUBO problem P with $\tau = 3$ for the graphs with 34 and 50 nodes after 5 and 2 runs respectively and returns the global minimum for the QUBO for other graphs after 1 run.*
- The graph $BA(50, 5)$ is the largest random graph considered on the QA. It is a dense graph and as τ increases the QUBO, Q becomes more dense and challenging to handle on the D-Wave quantum annealer. We observed no match between the Tabu QBSolv global minimum energy and the minimum energy of QA solution after 100 runs of the problem P with $\tau = 5$ on the D-Wave 2000Q. However, after performing a greedy steepest descent post-processing on the QA solution resulted in a solution that matched the Tabu QBSolv solution and that worked usually on the 1st run. This is not surprising as we

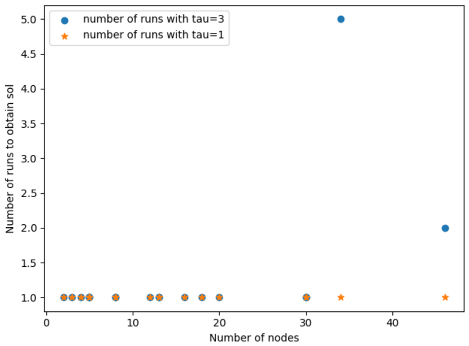

Fig 1. Number of runs plot ($\tau = 1$ and $\tau = 3$). *A plot of the number of nodes against the number of runs to obtain the optimal solution to the QUBO problem P, on the D-Wave 2000Q.*

recall that the D-Wave results are often approximate solutions and not always exact but can be improved by following it with post-processing methods (in this special case, running a greedy steepest descent was helpful). It is interesting to note that solving this same problem with simulated annealing yielded the result after 1 run. To ensure the result for the Barabasi Albert graph, BA(50,5,seed=7), in the table is reproducible we set the seed of the generator to a number (7 in this case to correspond to previous result for BA(50,5) with $\tau = 1, 3$).

- We have deleted the Scipy results presented earlier since that implementation has no significant relationship to our main objective of finding the top τ highest EC nodes.

4. ***Minor points:***
- Text refers to section numbers (e.g., "Section 2") but the sections do not have numbers - Figures are listed in the paper but appear at the end - First sentence in Conclusion is not clear: "We have formulated and shown a QUBO problem for .... is possible".

- *In the next section, we present a construction of a QUBO formulation for identifying a fraction of the most important nodes of the graph by the EC measure to be implemented on quantum computing devices. We then present the software tools, implementations and results obtained from the classical and quantum computers such as the D-Wave 2000Q and IBM-Q. We follow this up with a definition of node ranks from the results obtained from solving the QUBO on the D-Wave 2000Q and provide discussions on the conclusions derived from the results. Finally, we provide a summary of the results and conclude with*

suggestions of future directions to be considered.

- We paraphrase the first sentence in conclusion as *We have formulated and empirically shown the ground state of the QUBO problem P identifies the top most important node in a graph based on the EC measure.*

Reviewer 2

This is an interesting work. Honestly, I am not familiar to the topic of centrality and its application in graph. In this sense, the manuscript gives a brief and sound overview. The structure of the paper looks reasonable. My only comment is that there is a lack of the discussion regarding the noise during the simulations. A suggested revision can be focused on

1. ***presenting/comparing the data/results from both noise-free and noise-model simulator, as well as how both of them are compared to the data from real devices,***

    - An examination of the computational timings of the results from both noise-free and noise-model simulators favored the classical solvers, Numpy Minimum Eigensolver and tabu qbsolv, over quantum solvers, QA and QAOA, in generating results for the QUBO problem for $n \leq 50$ (see Fig 2). The time plots in Fig 2 show that as the number of nodes (n) increases, the total wall clock time spent by the quantum simulators to return a solution increases rapidly compared to the time spent by the classical solvers and the power iterative method employed by NetworkX. The observed relationship is approximately quadratic in n. The service time is the total wall clock time for a quantum machine instruction (QMI) to be sent to the D-Wave system, execute on the D-Wave system, and return a solution. It can be obtained by computing the time difference between the *start time* and *end time* for each QMI [2]. QPU access time is the time to execute a single QMI on the QPU and is composed of the QPU programming and sampling time. QPU programming time is basically a measure of the time taken to initialize the problem onto the QPU whereas the QPU sampling time is typically a measure of the time taken to actually execute the problem on the QPU. The QPU sampling time is made up of multiple anneal and readout times (taken to perform and read samples from the QPU) as well as the thermalization (time taken for the QPU to recover it's initial temperature) [2,3]. The QAOA time in Fig. 2 a is calculated here as the total time taken

to build (model time) and submit the QUBO to the IBM-Q QASM simulator plus the time taken to receive a solution.

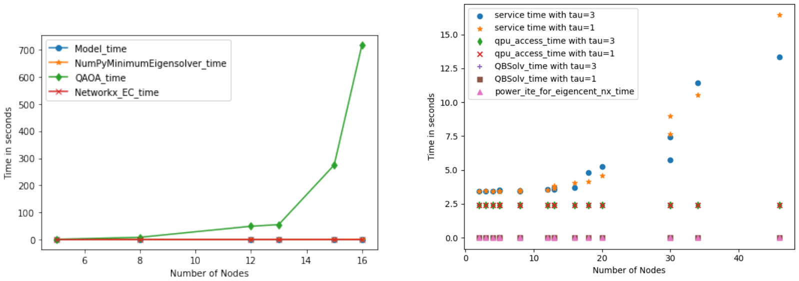

(a) Times from IBM-Q ($\tau = 1$) **(b)** Times from D-Wave 2000Q ($\tau = 1$ and $\tau = 3$)

Fig 2. Time plots. A plot of the number of nodes against the time in seconds taken to solve the QUBO problem P on (a) IBM-Q using (1) the classical exact numpy minimum eigensolver, (2) the QAOA algorithm on a QASM simulator, and using the power iterative method in NetworkX to solve the EC problem, and on (b) the D-Wave 2000Q using (1) the classical qbsolv solver which employs the tabu algorithm on the D-Wave system, (2) the QA algorithm and using the power iterative method in NetworkX to solve the EC problem.

Note that Fig. 2 here is Fig. 8 in the revised paper.

2. ***if the noise is overwhelming and altering the some observations, then is there any error-mitigation can be applied to improve the results.***

   - Error-mitigation techniques exist and can be applied to improve the results whenever there is an overwhelming noise affecting the performance. One of the methods we employed is the extended J range which involves the setting of a strong chain strength in defining a minor embedding [4]. This technique was employed to prevent broken chains and their negative effect on the D-Wave output. For our problem, low chain strength implied more broken chains. However after increasing the chain strength to the maximum (1000) at the time, we were able to obtain the desired global optima. Other techniques used include increasing the number of reads, annealing time and number of samples which have a positive influence on the probability of obtaining the optimal solution [5]. Another error-mitigation method is increasing the spin-reversal (Gauge) transforms, a technique that controls the influence of biases with a side effect of an increase in total run time of the problem [5]. By default,

the D-Wave system also uses a drift correction technique every hour to correct drifts [6]. The graph $BA(50,5)$ is the largest random graph considered on the D-Wave system. It is a dense graph and as τ increases the QUBO, Q becomes more dense and challenging to handle on the D-Wave quantum annealer. We observed no match between the global minimum energy of the Tabu QBSolv result and the minimum energy of QA result after 100 runs of the problem P with $\tau = 5$ on D-Wave 2000Q. However, after performing a greedy steepest descent post-processing on the QA solution resulted in a solution that matched the Tabu QBSolv solution and that worked usually on the 1st run. This is not surprising as we recall that D-Wave results are often approximate solutions and not always exact but can be improved by following it with post-processing methods (in this special case, running a greedy steepest descent was helpful). It is interesting to note that solving this same problem with simulated annealing yielded the solution after 1 run.

References

1. Childs AM, Goldstone J. Spatial search by quantum walk. Phys Rev A. 2004;70:022314. doi:10.1103/PhysRevA.70.022314.
2. Operation and Timing; 2022. `https://docs.dwavesys.com/docs/latest/c_qpu_timing.html`.
3. Breakdown of QPU Access Time; 2020. `https://docs.dwavesys.com/docs/latest/c_timing_2.html`.
4. Error-Correction Features; 2022. `https://docs.dwavesys.com/docs/latest/c_qpu_error_correction.html`.
5. QPU Solvers: Configuration; 2022. `https://docs.dwavesys.com/docs/latest/handbook_qpu.html`.
6. Error Sources for Problem Representation; 2022. `https://docs.dwavesys.com/docs/latest/c_qpu_ice.html#example-of-ice-effects-on-solution-quality`.
